# Exploring Potential Gender-Based Disparities in Referral for Transplant, Activation on the Waitlist and Kidney Transplantation in a Canadian Cohort

**DOI:** 10.1016/j.ekir.2024.04.039

**Published:** 2024-04-25

**Authors:** Amanda J. Vinson, Aran Thanamayooran, Karthik K. Tennankore, Bethany J. Foster

**Affiliations:** 1 Division of Nephrology, Department of Medicine, Dalhousie University, Halifax, Nova Scotia, Canada; 2 Nova Scotia Health, Halifax, Nova Scotia, Canada; 3 MOTP Research Program, Nova Scotia Health, Halifax, Nova Scotia, Canada; 4 Research Institute of the McGill University Health Center, Montréal, Quebec, Canada; 5 Department of Epidemiology, Biostatistics, and Occupational Health, McGill University, Montréal, Quebec, Canada; 6 Department of Pediatrics, Division of Nephrology, McGill University Faculty of Medicine, Quebec, Canada

**Keywords:** Canada, disparity, gender, inequity, kidney transplant, referral

## Abstract

**Introduction:**

In the United States, women are less likely to be referred, activated on the waitlist, or undergo kidney transplant (KT) than men; contemporary Canadian data regarding access to transplant for women are lacking.

**Methods:**

Among patients initiating dialysis in Nova Scotia (NS), Canada from 2010 to 2020, we examined the association of candidate gender with overall access to KT, including the following: (i) odds of transplant referral within 1 year of dialysis initiation, (ii) odds of activation on the transplant waitlist (if referred), and (iii) time-to-transplantation (if activated) using logistic regression or Cox proportional hazards models as appropriate.

**Results:**

Among 749 patients deemed potentially eligible for transplant, women had lower transplant rates than men (adjusted hazard ratio [aHR]: 0.53, 95% confidence interval [CI]: 0.36–0.78); this was amplified among patients aged >60 years (aHR: 0.25, 95% CI: 0.09–0.69). Compared with men, women had a lower adjusted odds of transplant referral (adjusted odds ratio [aOR]: 0.57, 95% CI: 0.35–0.93) by 1 year after dialysis initiation. Among those referred, women had lower odds of waitlist activation than men (aOR: 0.58, 95% CI: 0.30–1.11); and among those activated, women had lower hazard of KT (aHR: 0.74, 95% CI: 0.51–1.09), though these differences were not statistically significant. Women in NS experience lower overall access to transplant, including less referral, activation and KT compared with men.

**Conclusion:**

Gender-based barriers to any of (or in this case each of) referral, activation, or transplantation result in inequities in access; identification of disparities at these critical decision points is an important first step toward ensuring equal access for all.

## Graphical abstract

**Figure:**
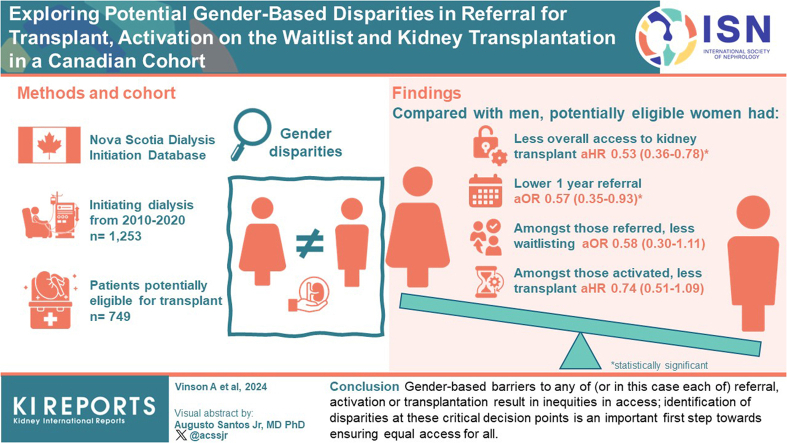

In the US, compared with men, women are less likely to be referred for KT, activated on the waitlist, and to undergo transplantation.^1^, ^2^, ^3^ The reasons for this are unknown. Factors that may contribute to these disparities include sex-based and/or gender-based differences in medical suitability for transplant, comorbidity, frailty, socioeconomic and/or sociocultural barriers, provider bias, and candidate self-selection or self-perceived eligibility. Contemporary Canadian data on gender disparities in access to KT are lacking. It is inappropriate to assume that American data can be generalized to Canada, a country with universal health care and distinct criteria for waitlist eligibility. In the US, gender-based differences in access to transplantation may be confounded by gender-based differences in socioeconomic status,^4^ a factor which may be less impactful in the Canadian health care setting. An analysis of referral practices for KT in Canada from 2010 to 2013 demonstrated significant variation in referral rates between provinces.^5^ Overall, females had a 12% lower hazard for KT referral than males, except in a subcohort restricted to those aged <60 years where there was no difference by sex. Sex disparity in kidney transplantation was not explored. Another Canadian study showed fewer females than males were referred for transplantation, but the study did not account for the lower rates of end-stage kidney disease (ESKD) in females than males.^6^ Finally, 2 previous Canadian studies (1981–1996 and 1990–1998) showed significantly lower transplant rates in females than males with ESKD.^7^^,^^8^ However, reasons for this difference (i.e. biases in referral, differences in waitlisting, and time from waitlist activation to transplant) were not examined.

To date, a granular examination of the 3 major steps at which sex-based and/or gender-based inequities may occur (referral, waitlist activation, and transplantation) has not been performed in a Canadian population. In this study, we examine overall access to transplant for women versus men, and at each of these steps separately so that potential gender-based inequities can be identified and subsequently addressed. We hypothesize that women are disadvantaged relative to men across the spectrum of access to KT in Canada, with lower rates in women than men for each of referral, waitlist activation, and transplant.

## Methods

### Data Sources and Study Population

Data were combined using the Nova Scotia Dialysis Initiation Database,^9^^,^^10^ which captures data on all patients with ESKD in NS; and the Atlantic Canada Multi-Organ Transplant Program database, which captures all patients referred for transplant, activated on the transplant waitlist, and ultimately transplanted in Atlantic Canada (Supplementary Figure S1). Among patients initiating dialysis between 2010 and 2020, we determined what percentage of eligible men and women in NS were referred, activated, and transplanted. Patient level data at the time of dialysis initiation exist in the Nova Scotia Dialysis Initiation Database for all Nova Scotians with ESKD, including those who were never referred for kidney transplantation. Nonreferred patients had their charts abstracted (blinded to patient gender) and eligibility for referral was determined based on existing Canadian guidelines.^11^^,^^12^ Patients were only considered ineligible if they were identified as having an absolute contraindication to transplantation: aged ≥76 years, body mass index >40 kg/m^2^; dementia; hemiplegia; severe untreated cardiovascular, peripheral vascular, or respiratory disease; active malignancy; nonadherence; and patients reporting they “did not want a transplant,” in keeping with general Atlantic Canadian referral practices. Eligibility for transplant in NS is determined by renal waitlist committee expert opinion. Nonreferred patients who did not have an absolute contraindication to transplantation at the time of ESKD had their charts formally reviewed by 2 members of the renal waitlist committee (blinded to patient gender) to determine if they were potentially eligible for referral, and discordant opinions regarding eligibility were decided by a third member.^11^ To be considered eligible for activation, a patient must have been referred for transplant, and to be considered eligible for transplant a patient must have been active on the waitlist (or approved for transplant in the setting of a living donor). For the sake of this study, approval for transplant and waitlist activation are considered synonymous.

### Analysis

#### Overall Access to Transplantation

Among all potentially eligible patients (lacking an absolute contraindication to transplant), we used multivariable Cox proportional hazards models to examine the independent association of candidate gender with kidney transplantation. Models were adjusted for potential confounders, including patient body mass index, age, race (White, Black, Other), dialysis status (preemptive, hemodialysis, or home therapy (hemodialysis or peritoneal dialysis), cause of ESKD (polycystic kidney disease, diabetes, glomerulonephritis, and other), pre-ESKD follow-up (nephrologist, other specialist, family physician, or no follow-up), smoking status, and comorbidity burden (cardiac disease, diabetes, peripheral vascular disease, and malignancy history). Models examining time to transplant were also adjsuted for panel reactive antibody status. Time zero was the date of dialysis start (wait-time based prioritization for transplant in NS includes only wait-time accrued after dialysis start), with a time of 0.001 days for patients transplanted preemptively (Supplementary Figure S2).

#### Referral, Activation, and Transplantation Steps

For men and women separately, we determined the median time from: (i) dialysis start to referral and (ii) dialysis start to transplantation. Unfortunately, activation date is not well-captured because patient wait time is backdated to dialysis start date functionally (and in our registry); therefore, time from referral to activation is not reliable for patients referred after dialysis start, nor is time from actual waitlist activation to transplant. The association of candidate gender with the following: (i) the odds of transplant referral within 1 year of dialysis initiation, (ii) the odds of activation on the transplant waitlist (among those referred), and (iii) time-to-transplantation (among those activated), were compared using logistic regression or Cox proportional hazards models as appropriate, adjusted for the covariates listed above.

In secondary analyses, we examined activation among all potentially eligible patients with ESKD (not just those referred), and transplantation among all patients referred (not just those activated). We also examined time-to-referral using multivariable Cox proportional hazards models with time zero being the date of dialysis initiation (with a time of 0.001 days for those referred and/or transplanted preemptively) (Supplementary Figure S2).

#### Gender-Specific Analyses

If a gender-based disparity was identified in any of referral, activation, or transplantation, specific barriers were sought by stratifying by gender and using multivariable logistic regression or Cox proportional hazards models as appropriate, including patient demographics, disease characteristics, and comorbidities to identify factors independently associated with nonreferral, nonactivation, or longer time-to-transplant for each gender.

#### Sensitivity Analyses

In sensitivity analyses, we examined the following:

1. The association of candidate gender with the odds of 2-year transplant referral (rather than 1 year).
2. Proportions of men and women referred for transplant preemptively.
3. Live versus deceased donor KT rates by candidate gender.
4. Gender-differences in adjusted referral, activation, and transplant rates (as for our primary analysis) in a cohort of all patients (not just those lacking an absolute contraindication to transplant).
5. The adjusted association of candidate gender with the odds of referral, activation, and transplant (primary analyses) in younger (aged ≤60 years) and older (aged >60 years) patients.
6. The unadjusted association of gender with each of transplant referral, activation, and transplant rates among patients lacking an absolute contraindication to transplant.
7. Time-to-transplant among the following: (i) all potentially eligible patients, (ii) those referred, and (iii) those activated on the waitlist; was examined accounting for the competing risk of death using a multivariable Fine and Gray competing risk subdistribution hazard model.^13^

Complete case analysis was performed; however variables with missingness >10% were treated with an indicator variable for missing values to avoid substantial loss of data. Patients were censored on the date of last follow-up (December 31, 2021) allowing for a minimum of 1 year follow-up for all patients.

## Results

### Overall Rates of Referral, Activation, and Transplantation

From 2010 to 2020, 1253 patients were identified as having ESKD (796 [63.5%] men and 457 [36.5%] women); 504 (40.2%) were deemed ineligible for transplant due to the presence of 1 or more absolute contraindications at the time of dialysis start. Of the patients, 411 (32.8% overall; 54.9% of those potentially eligible) were referred for transplant within 1 year of dialysis initiation; another 54 were referred between 1 and 2 years postdialysis initiation. A total of 498 patients were referred at any point during the study period. Of the patients, 291 (23.2% overall; 58.4% of those referred) were activated on the transplant waitlist and 226 were transplanted (18.0% overall; 77.7% of those activated) (Supplementary Figure S3).

### Gender-Differences in Patient Case-Mix

Baseline characteristics of all patients initiating dialysis over the study period, by patient gender, are shown in Table 1. Cardiac disease was more common in men than in women (33.3% vs. 22.8%) and women had more polycystic kidney disease (9.4% vs. 5.3%), and higher maximum panel reactive antibody (median 8.5% [interquartile range, IQR: 0–72] vs. 0% [IQR: 0–5.5]). There were no other significant differences by gender.

**Table 1:** Baseline characteristics

Variable	Men	Women
ESKD	796 (63.5%)	457 (36.5%)
Absolute contraindication (at ESKD)	316 (39.7%)	188 (41.1%)
Nonadherence	22 (7.0%)	15 (8.0%)
Patient does not want transplant	5 (1.6%)	6 (3.2%)
BMI >40	53 (16.8%)	48 (25.5%)
Severe respiratory diseasexye	2 (0.6%)	2 (1.1%)
Dementia	9 (2.9%)	4 (2.1%)
Active malignancy	15 (4.8%)	3 (1.6%)
Severe peripheral vascular disease	24 (7.6%)	8 (4.3%)
Severe/untreated cardiac disease	38 (12.0%)	28 (14.9%)
Aged ≥76 yr	148 (46.8%)	74 (39.4%)
No absolute Contraindication	480 (40.3%)	269 (38.9%)
BMI >30	224 (28.1%)	137 (30.0%)
Missing	213 (26.8%)	131 (28.7%)
Dialysis type		
Preemptive	108 (13.6%)	62 (13.6%)
Hemodialysis (in-center)	455 (57.2%)	263 (57.6%)
Home therapy (PD/HD)	108 (13.6%)	60 (13.1%)
Missing	125 (15.7%)	72 (15.8%)
Age, yr		
<50	139 (17.5%)	87 (19.0%)
50–60	162 (20.4%)	101 (22.1%)
>60	495 (62.2%)	269 (58.9%)
Race		
White	589 (74.0%)	326 (71.3%)
Black	30 (3.8%)	28 (6.1%)
Other	156 (19.6%)	85 (18.6%)
Missing	21 (2.6%)	18 (3.9%)
Cardiac disease	265 (33.3%)	104 (22.8%)
Peripheral vascular disease	120 (15.1%)	45 (9.9%)
Missing	93 (11.7%)	50 (10.9%)
Diabetes	299 (37.6%)	166 (36.3%)
Missing	246 (30.9%)	141 (30.9%)
Cancer history	42 (5.3%)	25 (5.5%)
Missing	249 (31.3%)	145 (31.7%)
Smoker	81 (10.2%)	46 (10.1%)
Missing	168 (21.1%)	92 (20.1%)
Cause of ESKD		
PCKD	42 (5.3%)	43 (9.4%)
Diabetes	247 (31.0%)	126 (27.6%)
GN	104 (13.1%)	61 (13.4%)
Other/unknown	403 (50.6%)	227 (49.7%)
Pre-ESKD follow-up		
Nephrologist	178 (22.4%)	93 (20.4%)
Specialist	214 (26.9%)	129 (28.2%)
Office/Clinic	49 (6.2%)	27 (5.9%)
No follow-up	96 (12.1%)	67 (14.7%)
Unknown	259 (32.5%)	141 (30.9%)
Max cumulative PRA	0 (0, 5.5)	8.5 (0, 72)
>25%	47 (5.9%)	61 (13.4%)
Missing^a^	504 (63.3%)	313 (68.5%)

BMI, body mass index; ESKD, end-stage kidney disease; GN, glomerulonephritis; HD, hemodialysis; PCKD, polycystic kidney disease; PD, peritoneal dialysis; PRA panel reactive antibody.

a Missingness for PRA is reported for the entire cohort (including patients not referred for transplant). Missingness among patients activated on the waitlist was 3.8%

The proportion of patients with an absolute contraindication to transplant was similar for men and women (39.7% vs. 41.1%, *P* value = 0.617). Reasons for absolute contraindications are shown in Table 1. There was no difference by gender in the odds of having an absolute contraindication (aOR: 1.06, 95% CI: 0.84–1.34 in women vs. men).

### Overall Gender Differences in Transplant Rates

**Figure 1:**
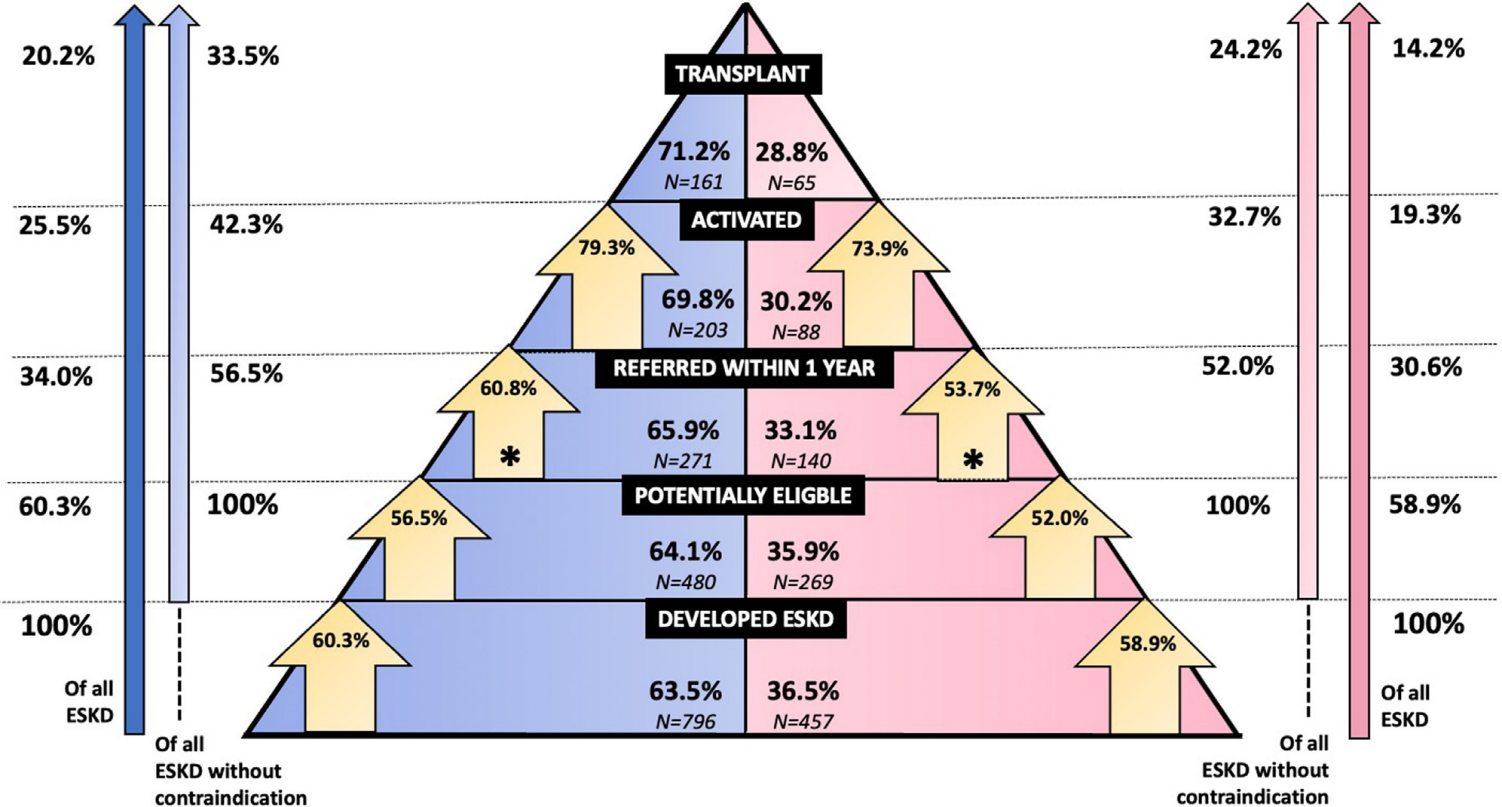
Gender differences in progression along the continuum from end-stage kidney disease to transplant. ESKD, end-stage kidney disease. (Blue: men, Pink: women).

**Figure 2:**
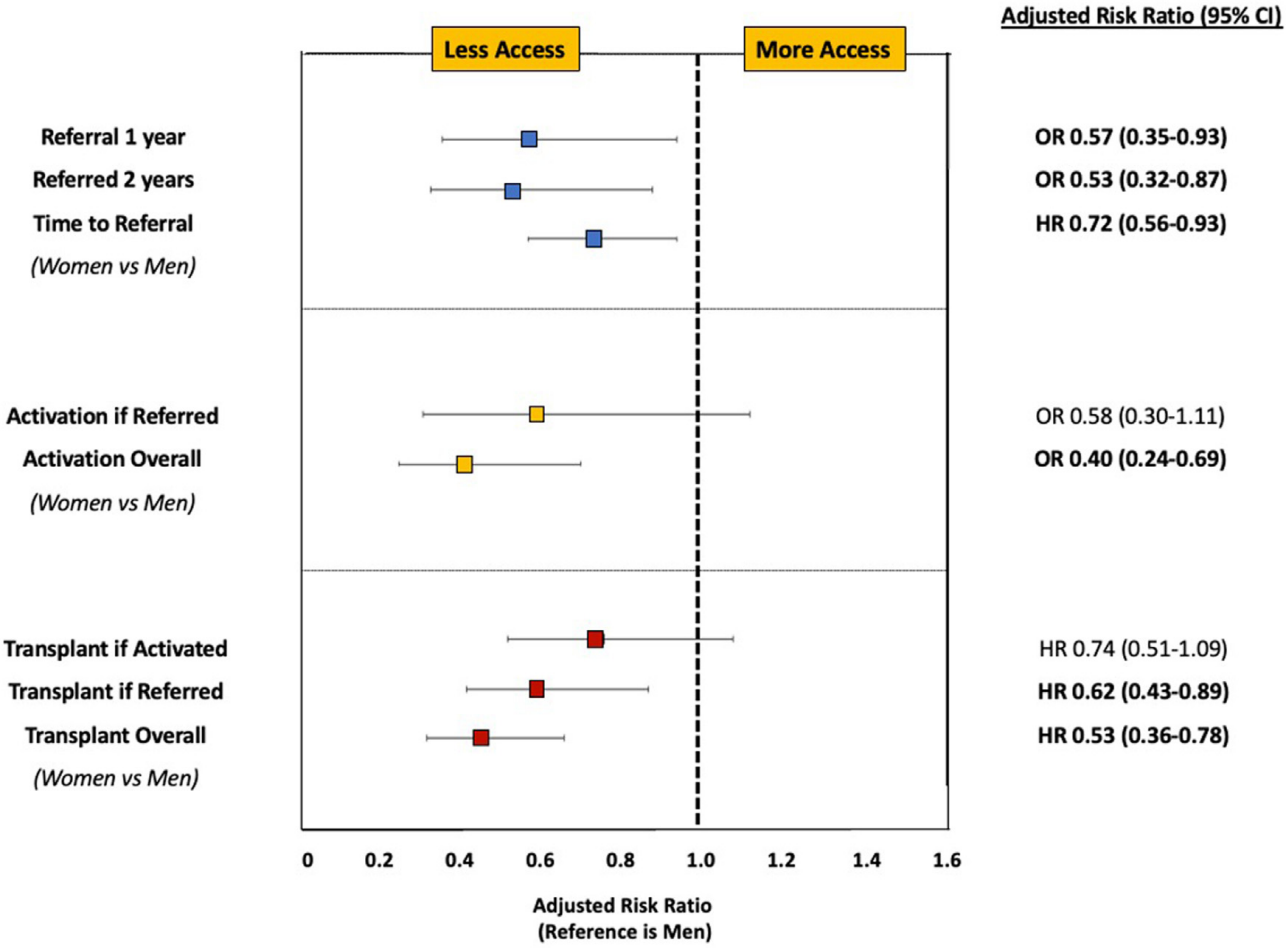
Multivariable models examining gender difference in referral, activation, and transplantation among potentially eligible patients. CI, confidence interval; HR, hazard ratio; OR, odds ratio.

Among all patients with ESKD, 20.2% of males and 14.2% of females underwent KT. This increased to 33.5% of males and 24.2% of females after excluding patients deemed to have an absolute contraindication as defined in our study (Figure 1). Among potentially eligible patients, transplantation rates were substantially lower in women than men (aHR: 0.53, 95% CI: 0.36–0.78) (Figure 2). Among those transplanted, median time from dialysis start to transplant was 1.69 years (IQR: 0.83–2.80) in men and 1.93 years (IQR: 1.02–3.07) in women (*P* value = 0.34).

### Referral

Of the patients, 271 men and 140 women (56.5% and 52.0% of potentially eligibly patients, respectively) were referred for transplant within 1 year of dialysis initiation. Compared with men, women without an absolute contraindication to kidney transplantation had significantly lower odds of transplant referral (aOR: 0.57, 95% CI: 0.35–0.93) within 1 year of dialysis initiation (Figure 2 and Supplementary Table S1). Among patients referred for transplant, the median time from dialysis initiation to referral was 22 days (IQR: 0.00–492) in men and 60 days (IQR: 0.00–4782) in women (*P* value = 0.96).

### Activation

Of those referred at any time during the study period, 203 men (60.8%) and 88 women (53.7%) were activated on the transplant waitlist (Figure 1). Referred women had lower odds of waitlist activation than men (aOR: 0.58, 95% CI: 0.30–1.11), though this was not statistically significant (Figure 2 and Supplementary Table S1). When examining gender differences in waitlist activation among all potentially eligible patients (not just those referred), women had significantly lower waitlist activation than men (aOR: 0.40, 95% CI: 0.24–0.69).

### Transplantation

Among activated patients, 161 men and 65 women (79.3% and 73.9% of men and women on the waitlist, respectively) underwent KT. Among those activated, women had a lower hazard of transplant than men (aHR: 0.74, 95% CI: 0.51–1.09), but not significantly (Figure 2 and Supplementary Table S1). Among all referred patients, transplantation rates were significantly lower in women than men (aHR: 0.62, 95% CI: 0.43–0.89).

### Gender-Specific Analysis

Gender-specific analyses were limited by small sample sizes but are shown in Supplementary Table S2 for (A) 1 year referral, (B) waitlist activation among those referred, and (C) transplantation among those activated. Findings were generally similar.

### Sensitivity Analyses

When looking at 2-year referral rates, results were unchanged (aOR: 0.53, 95% CI: 0.32–0.87 for women vs. men). Of the 213 (63.8%) men and 106 (64.6%) women ultimately referred for KT, women had a lower adjusted odds of transplant referral prior to dialysis initiation than men (aOR: 0.55, 95% CI: 0.33–0.90) in keeping with the overall lower referral rates for women. Of those who underwent transplant, 54 (35.1%) men and 16 (25.4%) women received a living donor kidney (*P* value = 0.167). In multivariable models, patient gender did not significantly associate with the odds of receiving a kidney from a living donor (aOR: 0.99, 95% CI: 0.38–2.54), though the numbers were small.

**Figure 3:**
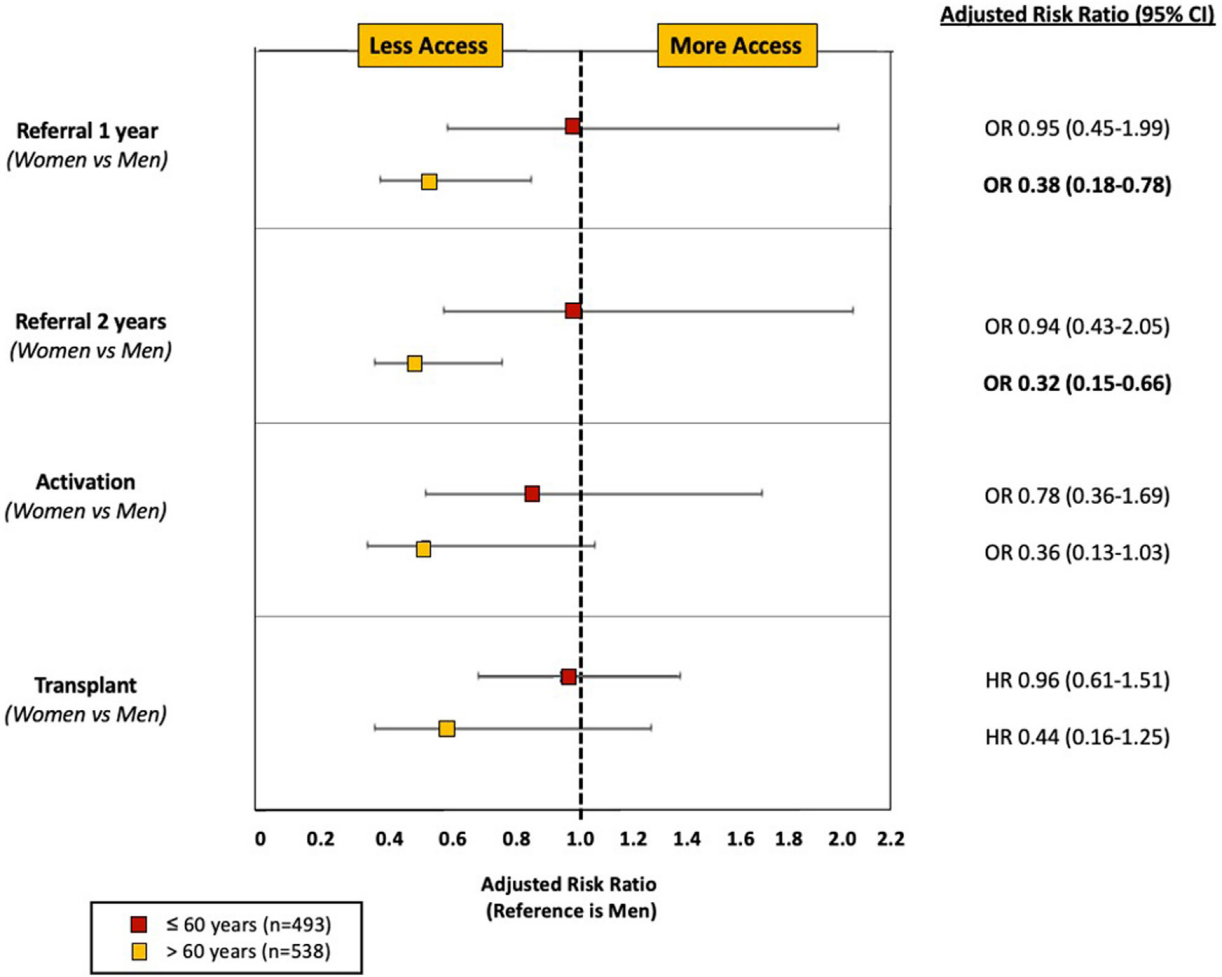
Multivariable models examining gender difference in referral, activation, and transplantation among potentially eligible patients, stratified by age. CI, confidence interval; ESKD, end-stage kidney disease; HR, hazard ratio; OR, odds ratio.

When the denominator of “eligible” patients was all patients with ESKD (rather than those lacking an absolute contraindication as defined in our current study), transplant referral rates remained lower among women than among men by 1 year after dialysis initiation (aOR: 0.54, 95% CI: 0.37–0.80) (Table 2). Finally, when stratified by age, there were no significant gender differences in referral rates, waitlist activation, or transplantation in patients aged ≤60 years (aOR: 0.95, 95% CI: 0.45–1.99 for 1 year referral; aOR: 0.78, 95% CI: 0.36–1.69 for activation; aHR: 0.96, 95% CI: 0.61–1.51 for transplantation); however, in older patients (aged >60 years), the gender differences were amplified for referral (aOR: 0.38, 95% CI: 0.18–0.78 at 1 year), activation (aOR: 0.36, 95% CI: 0.13–1.03), and transplantation (aHR: 0.44, 95% CI: 0.16–1.25), (Figure 3). An age-stratified analysis examining waitlist activation and transplantation among all potentially eligible patients is shown in Supplementary Figure S4. Among all eligible patients with ESKD, women had a lower adjusted hazard of receiving a KT that was magnified in the older cohort (aHR: 0.62, 95% CI: 0.40–0.97 for those aged ≤60 years; aHR: 0.25, 95% CI: 0.09–0.69 for those aged >60 years).

**Table 2:** Summarized adjusted models examining gender differences in referral, activation, and transplantation in potentially eligible and all patients with end-stage kidney disease

Variable	Odds ratio (95% CI) for referral at 1 yr	HR (95% CI) for referral	Activation among those referred (OR)	Transplantation among those activated (HR)
Adjusted^a^ among those without absolute contraindication Gender (W vs. M)^b^	0.57 (0.35–0.93)	0.72 (0.56–0.93)	0.58 (0.30–1.11)	0.74 (0.51–1.09)
Adjusted^a^ among all patients with ESKD Gender (W vs. M)	0.54 (0.37–0.80)	0.64 (0.51–0.81)		

CI, confidence interval; ESKD, end-stage kidney disease; HR, hazard ratio; M, men; OR, odds ratio; W, women.

a Adjusted for same covariates listed above.

b The primary analysis for comparison.

Unadjusted results are depicted in Supplementary Table S3. There was a trend toward less access to transplant for women than men that only met significance when examining overall rate of transplant (HR: 0.64, 95% CI: 0.46–0.87). The subdistribution HRs for time-to-transplant in all potentially eligible women versus men, among only those referred, and among only those active on the waitlist are shown in Supplementary Table S4 and were essentially unchanged from the primary analysis.

## Discussion

**Figure 4:**
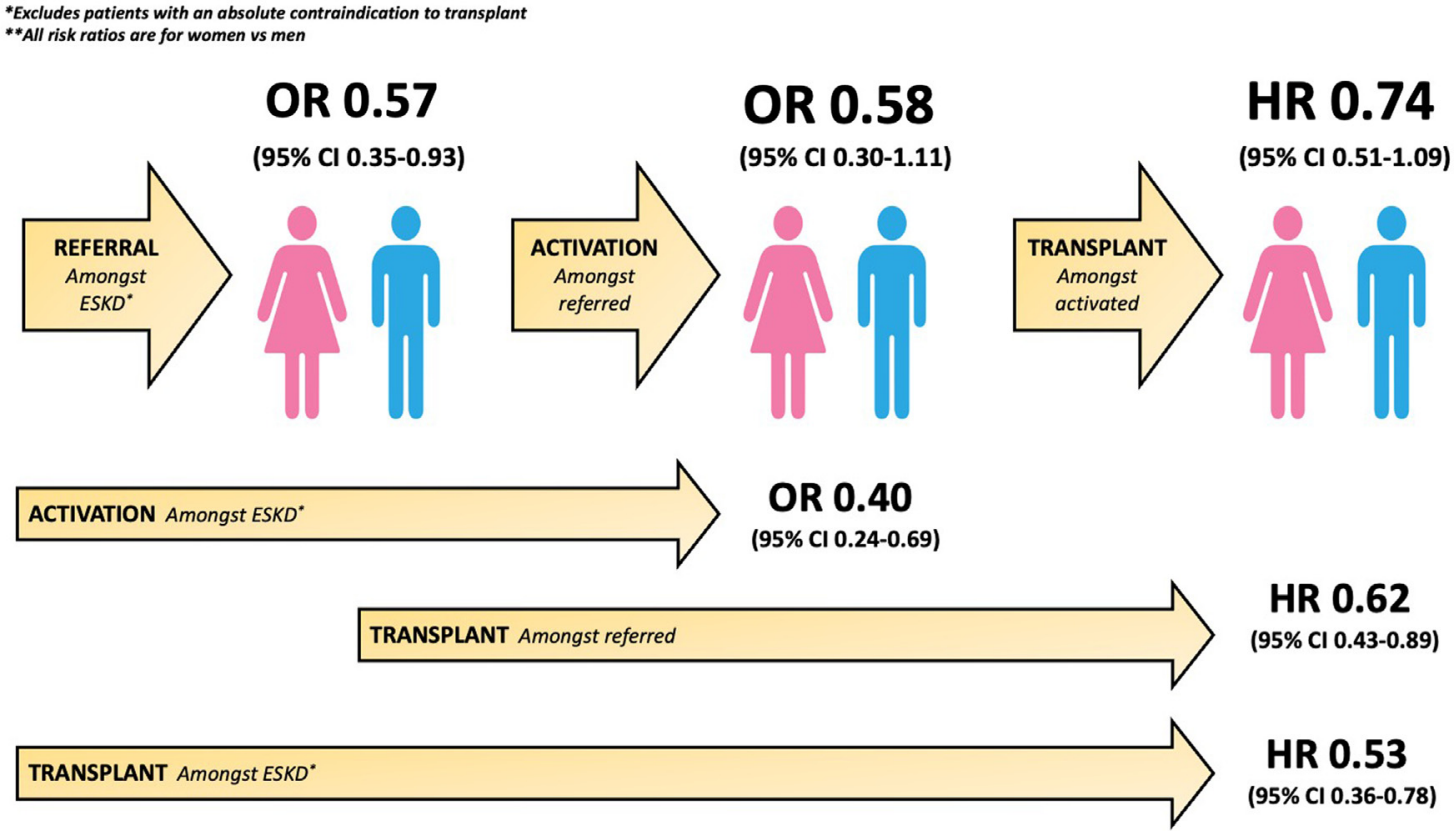
Summary of gender inequities along the continuum from end-stage kidney disease to transplantation. CI, confidence interval; ESKD, end-stage kidney disease; HR, hazard ratio; OR, odds ratio.

In this study, for the first time, we identify substantial gender-based inequities in referral, waitlist activation, and kidney transplantation in a Canadian cohort of potentially eligible patients with ESKD. As summarized in Figure 4, potentially eligible women had a 47% lower overall rate of transplant than men, with a 43% lower odds of transplant referral, 42% lower odds of waitlist activation (among those referred), and even among those activated, a 26% lower hazard of transplant. This was amplified in patents aged >60 years, where women had a 75% lower hazard for transplant than age-matched men overall, with 62% less referral, 64% less activation, and 56% less transplantation among those activated, than older men. Notably, there were no gender differences in referral rates among patients aged ≤60 years. Despite a trend toward less access for women than men, gender differences were not significant for waitlist activation or transplant in the overall or age restricted cohort. Therefore, although potentially eligible women appear to be disadvantaged along the entire spectrum from ESKD to transplant, the greatest barrier appears to be the significantly lower referral rates they face than men.

This is not the first study to identify gender disparities in access to kidney transplantation (with intersections noted for age, race, and obesity status).^14^^,^^15^ A recent analysis by the European Committee on Organ Transplantation of the Council of Europe demonstrated that in 2019, males outnumbered female KT recipients in 57 out of 62 surveyed countries across 6 continents.^16^ Notably, a major limitation of this and many other analyses, is that they did not account for baseline differences in ESKD prevalence, which is greater in males than females.^17^ Similarly, a 2022 scoping review examining inequities in access to organ transplant in the US demonstrated that women were less likely to be referred, evaluated, and added to the KT waitlist.^15^ Although Canadian data regarding gender equity in transplantation are more sparse, an earlier 2010 to 2013 pan-Canadian study demonstrated 12% lower adjusted transplant referral rates for women with ESKD than men.^5^ Interestingly however, in keeping with our findings, there were no gender differences in patients aged <60 years.^5^ However, the aim of this past study was to examine provincial variation in transplant referral rates, and potential reasons for these observed gender discrepancies, including the intersection with age, were not discussed. Likewise, in the US, women aged >45 years have less access to transplant than older men (59% lower for women than men aged >75 years), despite no gender differences in a subcohort of younger patients aged 18 to 45 years.^18^ The fact that gender differences in referral exist only among older patients might indicate differences in provider perception (or patient self-perception) of frailty among older individuals.^19^ Nonfrail women on hemodialysis have been shown to be misclassified by their health care providers as subjectively frail more frequently than nonfrail men.^20^ This may result in the perception that these women may experience poor outcomes on the waitlist or posttransplant, leading to a corresponding reduction in transplant referral, despite existing evidence that frailty and advanced age have less impact on survival in females than in males.^21^^,^^22^ Although the gender-specific analyses were limited by small numbers and results must therefore be interpreted with caution, we noted no difference in transplant access based on whether or not a patient had pre-ESKD nephrologist care. Notably however, we were unable to determine whether lack of a preexisting relationship with the health care system in general modified gender differences in accessing KT. This requires further study in a larger population, given the risk that without consistent prior patient follow-up, physician gestalt at the time of an acute event or at ESKD presentation takes precedence, with the potential for the perception that women more so than men are transplant ineligible, perhaps on the basis of informal bedside frailty judgments. The known challenges women face in terms of lower health literacy, self-efficacy, and self-advocacy,^19^^,^^23^^,^^24^ may be exacerbated when a trusting relationship with health care providers has not been previously established allowing time for such conversations to occur. Women have reported higher psychosocial and health-related concerns regarding KT^25^ and lack of consistent discussions with a medical provider before ESKD diagnosis may not allow these fears to be reasonably allayed. However, even once nephrology care is established, women with ESKD are still 45% more likely not to discuss KT with their health care providers than men, a disparity worsened still with advancing age.^26^

Of note, several earlier studies suggested that women experience lower waitlisting and poorer access to living donor transplantation than men; however, once waitlisted, they experience no difference in time-to-transplant.^18^^,^^27^ This is contrary to our current Canadian study, where access to transplant was lower for women at each step along the continuum (lower referral, with a strong trend toward lower waitlisting among those referred, and lower transplant among those waitlisted). It is likely that different mechanisms contribute to the sex and/or gender inequities observed for each event leading from ESKD to transplant. Many studies highlight the barriers female recipients face in accessing transplant due to pregnancy-induced sensitization,^28^^,^^29^ though differences in panel reactive antibody status should not influence upstream events such as referral or activation. Possible gender differences contributing to inequities in transplant referral have been proposed, including differences in patient eligibility (including comorbidity and age distribution), patient self-efficacy or self-advocacy, health care provider bias (subconscious or otherwise) and differential perception of patient frailty.^19^ Desire for a transplant may also differ between men and women; although numbers were small, in the current study, women were twice as likely as men to not want a transplant (3.2% vs. 1.6%). Importantly, 82.5% of referred patients were referred within the first year after dialysis initiation (81.1% for men and 85.4% for women), with few patients bypassed for referral in the early postdialysis period referred subsequently. An earlier US analysis of racial inequities in access to transplantation showed that compared with White patients, Black patients had 65% lower upfront access to transplant in the first year after dialysis initiation; however, by 4 years this disparity had resolved.^30^ This however does not appear to be the case for gender-based disparities; reasons why we do not see this “catch-up” in transplant referral for women requires attention, though it suggests that it is not simply that women require more time-to-consider and agree to referral than men.

Once referred, women may be less likely to navigate and complete the medical evaluation required for potential activation on the transplant waitlist.^2^ Nephrologists perceive that women face increased sociocultural barriers to transplant on account of greater caregiving responsibilities, social disadvantage, stereotyping, and stigma, than experienced by men,^31^ a perception which in and of itself may influence provider referral patterns.^32^ In addition, completion of pretransplant evaluation is influenced by degree of instrumental social support networks,^33^ with men generally experiencing greater support than women.^34^ Indeed, an earlier study in the Southeastern US demonstrated that referred women were 6% less likely than referred men to initiate their transplant evaluation.^35^ Collectively, these factors may manifest as a greater challenge to initiate and complete all necessary work-up including the multidimensional risk assessment required to ascertain transplant eligibility, and it is hypothesized that incomplete work-up may largely contribute to this observed difference. Even among those who complete the transplant evaluation process, women may be perceived by care providers to be less eligible for transplant than men; women have been shown to be less likely to be activated on the waitlist, even with similar or lower comorbidity burden.^18^^,^^36^ Similarly, obesity (body mass index >25 to 39 kg/m^2^) has been associated with a lower likelihood of activation on the transplant waitlist in females, but not males.^14^ Finally, some of the prolonged time-to-transplant in females versus males active on the waitlist may relate to pregnancy-induced sensitization; however, even after accounting for panel reactive antibody status, we showed longer time-to-transplant for waitlisted females than males in the current study. Women are known to be more forthcoming than men when reporting both major and minor medical concerns;^37^ whether this reporting bias leads to more frequent waitlist holds and longer duration of holds among waitlisted women than men is unknown.

Although this is the first study to examine gender differences in access to kidney transplantation in Canada (a country with socialized health care) along the entire continuum from ESKD to transplant, there are important limitations. First, we do not have data regarding transplant work-up initiation or completion rates; thus, it is unclear whether the gender differences in waitlist activation reflect differences in work-up completion, or actual differences in perceived eligibility by the renal waitlist committee. Having information regarding whether there are significant differences in time-to-waitlist completion (and gender differences in waitlist holds once activated) would contribute to the growing body of literature regarding barriers that women face in accessing a KT and is an area for future study. Another limitation is that absolute contraindications for our regional program may not be generalizable to other programs. For example, whereas all programs would consider active malignancy a contraindication to KT,^38^^,^^39^ many other programs do not consider body mass index or age thresholds when ascertaining transplant candidacy. In addition, this study examines referral rates in a single Canadian province, and therefore may not be generalizable to regions that comprise different patient case-mix. Given that there is likely some gender bias at each stage of the process (referral, activation, transplant), whereby women selected to proceed represent a healthier group than men selected to proceed, it is possible that our estimates represent underestimations of the true magnitude of gender differences. Furthermore, we did not have information regarding socioeconomic status, insurance provider (acknowledging Canada has socialized health care), or education status; all of which may differentially associate with gender and transplant access.^40^ Similarly, information regarding comorbidity severity was lacking, which may also influence decisions regarding transplant eligibility. Although it has been suggested that health care provider gender may influence decision making regarding transplant eligibility according to frailty or medical complexity,^41^ the treating physician’s gender was not available for this analysis. This study is 1 of the most granular analyses of gender disparities in access to transplant in that each patient chart was abstracted (blind to gender) to identify the presence of potential contraindications to KT; however, there is still much about physician gestalt and rationale behind nonreferral that was missing from the medical record. We were unable to differentiate between patients who were considered or approached for transplant referral but ultimately perceived by the approaching physician as being ineligible for reasons not captured in our database (including adherence or behavioral concerns, or subjective frailty) and therefore not formally referred, and those who were overlooked and never considered or approached. Whether women are more likely to be overlooked for transplant consideration completely, or approached but more likely to be deemed ineligible due to conscious or unconscious provider bias, is a point of future study. Finally, gender and gendered behaviors, including sociocultural gender norms, values, and identity are likely as, or more, contributory to these disparities in access than is biological sex. However, in this study, sex and gender were assumed to be concordant because patient self-reported gender was not available. In future studies, patients should be asked to report their gender, acknowledging that gender is a spectrum (not just man or woman) and may be nonbinary and fluid.^42^

In conclusion, this study demonstrates that compared with men, Atlantic Canadian women are disadvantaged along the entire continuum from ESKD to transplant, experiencing lower transplant referral (significantly so), waitlist activation, and eventual transplant. Further investigation is planned to better identify the exact reason for these pervasive disparities, so that sex-based and/or gender-based barriers can be challenged, and ultimately overcome. Randomized controlled trials examining educational initiatives to enhance racial (but not gender) equity in kidney transplantation have been successfully employed in the past.^43^^,^^44^ Future initiatives should focus on the creation of standardized assessment tools, patient engagement strategies, or patient and health care provider education programs, including those employed to minimize racial bias in transplant access, along the referral, waitlisting, and transplant continuum. Equity in access to transplantation is 1 of the cornerstones of organ allocation policy but can only be achieved when there is parity in referral, waitlist activation, and eventual transplant. Barriers for women to any of (or in this case each of) referral, activation, or transplantation will result in inequities in access; identification of disparities at these critical decision points is an important first step toward ensuring equal access for all.

## Disclosure

AJV has received consultancy funding from Paladin Labs Inc. and Takeda Pharmaceuticals. KKT has received fundings from Otsuka, Bayer, GSK, and Vifor. BJF is the chair of The Transplantation Society’s Women in Transplantation initiative. AT declared no competing interests.

## Supplementary Material

Supplementary File (PDF) **Figure S1.** Summary of data sources and approach to patient classification. **Figure S2.** Schematic of time intervals for access to transplant. Primary analysis treats referral at 1 year and waitlist activation as binary (yes/no) variables. (A) Transplant referral occurs after dialysis initiation and time-to-referral day 0 = dialysis initiation. Because waitlist activation is backdated to dialysis start, time-to-transplant day 0 = dialysis initiation date. (B) Preemptive transplant referral with dialysis initiation prior to transplant. In this case, time-to-referral is set to 0.001 days. Time-to-transplant day 0 = dialysis initiation date. (C) Preemptive transplant referral and preemptive transplant. In this case, both time-to-referral and time-to-transplant are set to 0.001 days. Because waitlist activation date is not available, there is no option to examine time from referral to waitlist activation. **Figure S3.** Flow diagram and cohort derivation. **Figure S4.** Multivariable models examining gender difference (women vs. men) in referral, activation, and transplantation among potentially eligible patients, those referred, and those waitlisted, stratified by age. **Table S1.** Multivariable logistic regression model for referral at 1 year, referral at 2 years, activation, and multivariable Cox proportional hazards model for time-to-transplantation (no absolute contraindication). **Table S2.** (A) Odds of referral at 1 year postdialysis initiation in women versus men. (B) Odds of waitlist activation among referred (at any time point) in women versus men. (C) Time-to-transplant among waitlist activated in women versus men. **Table S3.** Univariable logistic regression model for referral at 1 year, referral at 2 years, activation, and univariable Cox proportional hazards model for time-to-transplantation among potentially eligible patients. **Table S4.** Multivariable competing risk regression model (Fine and Gray) for time-to-transplantation among all potentially eligible patients, those referred, and those active on the waitlist. **STROBE Checklist.**

## Footnotes

Supplementary File (PDF)
**Figure S1.** Summary of data sources and approach to patient classification. **Figure S2.** Schematic of time intervals for access to transplant. Primary analysis treats referral at 1 year and waitlist activation as binary (yes/no) variables. (A) Transplant referral occurs after dialysis initiation and time-to-referral day 0 = dialysis initiation. Because waitlist activation is backdated to dialysis start, time-to-transplant day 0 = dialysis initiation date. (B) Preemptive transplant referral with dialysis initiation prior to transplant. In this case, time-to-referral is set to 0.001 days. Time-to-transplant day 0 = dialysis initiation date. (C) Preemptive transplant referral and preemptive transplant. In this case, both time-to-referral and time-to-transplant are set to 0.001 days. Because waitlist activation date is not available, there is no option to examine time from referral to waitlist activation. **Figure S3.** Flow diagram and cohort derivation. **Figure S4.** Multivariable models examining gender difference (women vs. men) in referral, activation, and transplantation among potentially eligible patients, those referred, and those waitlisted, stratified by age. **Table S1.** Multivariable logistic regression model for referral at 1 year, referral at 2 years, activation, and multivariable Cox proportional hazards model for time-to-transplantation (no absolute contraindication). **Table S2.** (A) Odds of referral at 1 year postdialysis initiation in women versus men. (B) Odds of waitlist activation among referred (at any time point) in women versus men. (C) Time-to-transplant among waitlist activated in women versus men. **Table S3.** Univariable logistic regression model for referral at 1 year, referral at 2 years, activation, and univariable Cox proportional hazards model for time-to-transplantation among potentially eligible patients. **Table S4.** Multivariable competing risk regression model (Fine and Gray) for time-to-transplantation among all potentially eligible patients, those referred, and those active on the waitlist.
**STROBE Checklist.**
