# Supplementary material for: Exploring Potential Gender-Based Disparities in Referral for Transplant, Activation on the Waitlist and Kidney Transplantation in a Canadian Cohort
Source: Kidney Int Rep. 2024 Apr 25;9(7):2157–67. doi: 10.1016/j.ekir.2024.04.039 (PMC11284405; doi:10.1016/j.ekir.2024.04.039)
Supplement: Supplementary File (PDF) — Figure S1. Summary of data sources and approach to patient classification. Figure S2. Schematic of time intervals for access to transplant. Primary analysis treats referral at 1 year and waitlist activation as binary (yes/no) variables. (A) Transplant referral occurs after dialysis initiation and time-to-referral day 0 = dialysis initiation. Because waitlist activation is backdated to dialysis start, time-to-transplant day 0 = dialysis initiation date. (B) Preemptive transplant referral with dialysis initiation prior to transplant. In this case, time-to-referral is set to 0.001 days. Time-to-transplant day 0 = dialysis initiation date. (C) Preemptive transplant referral and preemptive transplant. In this case, both time-to-referral and time-to-transplant are set to 0.001 days. Because waitlist activation date is not available, there is no option to examine time from referral to waitlist activation. Figure S3. Flow diagram and cohort derivation. Figure S4. Multivariable models examining gender difference (women vs. men) in referral, activation, and transplantation among potentially eligible patients, those referred, and those waitlisted, stratified by age. Table S1. Multivariable logistic regression model for referral at 1 year, referral at 2 years, activation, and multivariable Cox proportional hazards model for time-to-transplantation (no absolute contraindication). Table S2. (A) Odds of referral at 1 year postdialysis initiation in women versus men. (B) Odds of waitlist activation among referred (at any time point) in women versus men. (C) Time-to-transplant among waitlist activated in women versus men. Table S3. Univariable logistic regression model for referral at 1 year, referral at 2 years, activation, and univariable Cox proportional hazards model for time-to-transplantation among potentially eligible patients. Table S4. Multivariable competing risk regression model (Fine and Gray) for time-to-transplantation among all potentially eligible patients, those ref [file mmc1.pdf]

STROBE Statement—Checklist of items that should be included in reports of ***cohort studies***

	Item No	Recommendation	Page No
**Title and abstract**	1	(*a*) Indicate the study's design with a commonly used term in the title or the abstract<br>(*b*) Provide in the abstract an informative and balanced summary of what was done and what was found	1, 2
**Introduction**			
Background/rationale	2	Explain the scientific background and rationale for the investigation being reported	4,5
Objectives	3	State specific objectives, including any prespecified hypotheses	4,5
**Methods**			
Study design	4	Present key elements of study design early in the paper	6
Setting	5	Describe the setting, locations, and relevant dates, including periods of recruitment, exposure, follow-up, and data collection	5
Participants	6	(*a*) Give the eligibility criteria, and the sources and methods of selection of participants. Describe methods of follow-up<br>(*b*) For matched studies, give matching criteria and number of exposed and unexposed	5,6
Variables	7	Clearly define all outcomes, exposures, predictors, potential confounders, and effect modifiers. Give diagnostic criteria, if applicable	6
Data sources/ measurement	8*	For each variable of interest, give sources of data and details of methods of assessment (measurement). Describe comparability of assessment methods if there is more than one group	5,6
Bias	9	Describe any efforts to address potential sources of bias	5-8
Study size	10	Explain how the study size was arrived at	5
Quantitative variables	11	Explain how quantitative variables were handled in the analyses. If applicable, describe which groupings were chosen and why	6
Statistical methods	12	(*a*) Describe all statistical methods, including those used to control for confounding<br>(*b*) Describe any methods used to examine subgroups and interactions<br>(*c*) Explain how missing data were addressed<br>(*d*) If applicable, explain how loss to follow-up was addressed<br>(*e*) Describe any sensitivity analyses	6-8
**Results**			
Participants	13*	(a) Report numbers of individuals at each stage of study—eg numbers potentially eligible, examined for eligibility, confirmed eligible, included in the study, completing follow-up, and analysed<br>(b) Give reasons for non-participation at each stage<br>(c) Consider use of a flow diagram	8-10
Descriptive data	14*	(a) Give characteristics of study participants (eg demographic, clinical, social) and information on exposures and potential confounders<br>(b) Indicate number of participants with missing data for each variable of interest<br>(c) Summarise follow-up time (eg, average and total amount)	8-9
Outcome data	15*	Report numbers of outcome events or summary measures over time	8-10

Main results	16	(*a*) Give unadjusted estimates and, if applicable, confounder-adjusted estimates and their precision (eg, 95% confidence interval). Make clear which confounders were adjusted for and why they were included<br>(*b*) Report category boundaries when continuous variables were categorized<br>(*c*) If relevant, consider translating estimates of relative risk into absolute risk for a meaningful time period	8-10
Other analyses	17	Report other analyses done—eg analyses of subgroups and interactions, and sensitivity analyses	11,12
**Discussion**			
Key results	18	Summarise key results with reference to study objectives	12
Limitations	19	Discuss limitations of the study, taking into account sources of potential bias or imprecision. Discuss both direction and magnitude of any potential bias	16,17
Interpretation	20	Give a cautious overall interpretation of results considering objectives, limitations, multiplicity of analyses, results from similar studies, and other relevant evidence	17
Generalisability	21	Discuss the generalisability (external validity) of the study results	16,17
**Other information**			
Funding	22	Give the source of funding and the role of the funders for the present study and, if applicable, for the original study on which the present article is based	1

*Give information separately for exposed and unexposed groups.

Note: An Explanation and Elaboration article discusses each checklist item and gives methodological background and published examples of transparent reporting. The STROBE checklist is best used in conjunction with this article (freely available on the Web sites of PLoS Medicine at http://www.plosmedicine.org/, Annals of Internal Medicine at http://www.annals.org/, and Epidemiology at http://www.epidem.com/). Information on the STROBE Initiative is available at http://www.strobe-statement.org.

Supplemental Figure 1: Summary of Data Sources and Approach to Patient Classification

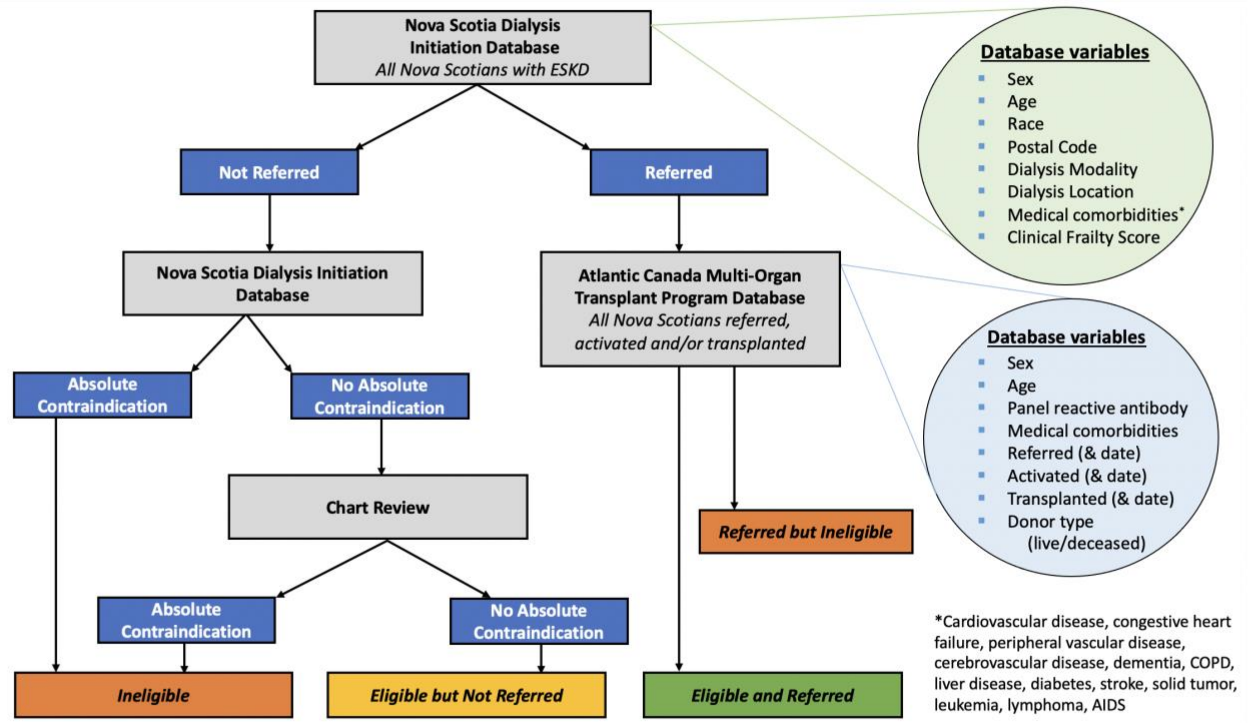

Supplemental Figure 2: Schematic of time intervals for access to transplant

Primary analysis treats referral at 1 year and waitlist activation as binary (yes/no) variables. ***A)*** *Transplant referral occurs after dialysis initiation and time to referral day 0 = dialysis initiation. Because waitlist activation is backdated to dialysis start, time to transplant day 0 = dialysis initiation date.* ***B)*** *Pre-emptive transplant referral with dialysis initiation prior to transplant. In this case time to referral is set to 0.001 days. Time to transplant day 0 = dialysis initiation date.* ***C)*** *Pre-emptive transplant referral and pre-emptive transplant. In this case both time to referral and time to transplant are set to 0.001 days. Because waitlist activation date is not available, there is no option to examine time from referral to waitlist activation.*

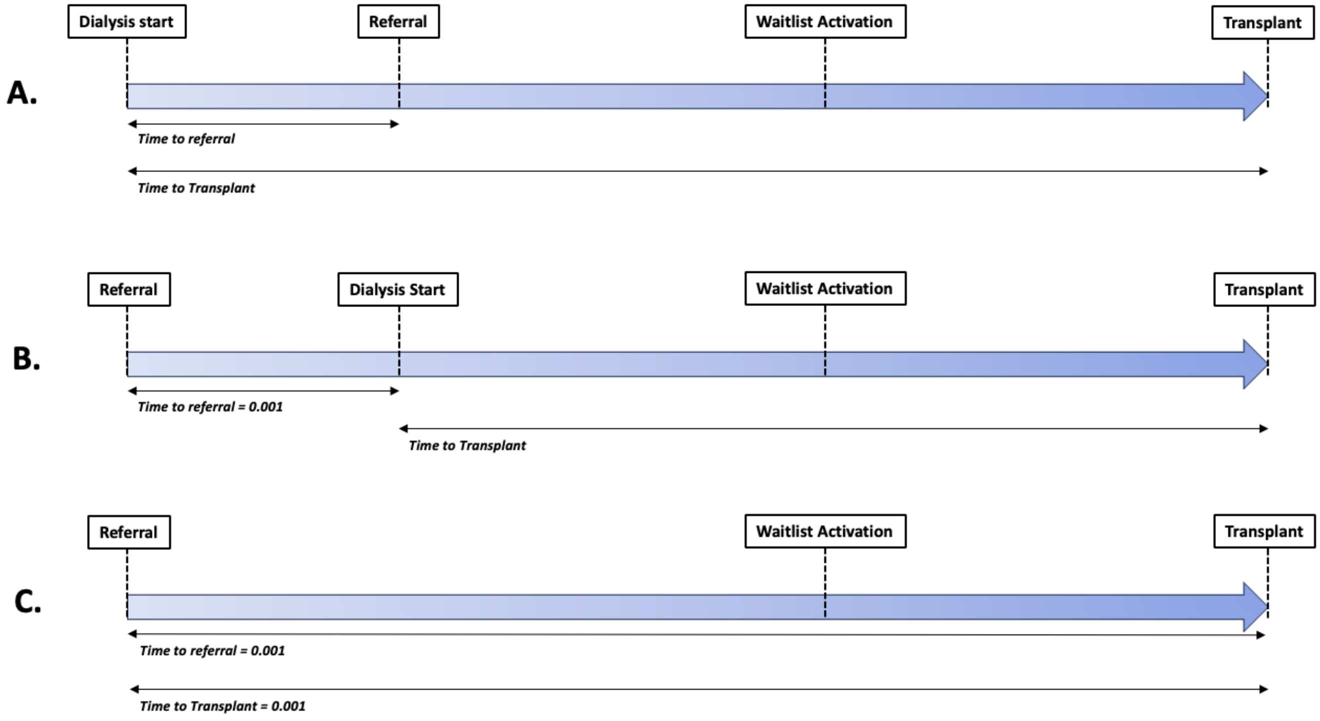

Supplemental Figure 3: Flow Diagram and Cohort Derivation

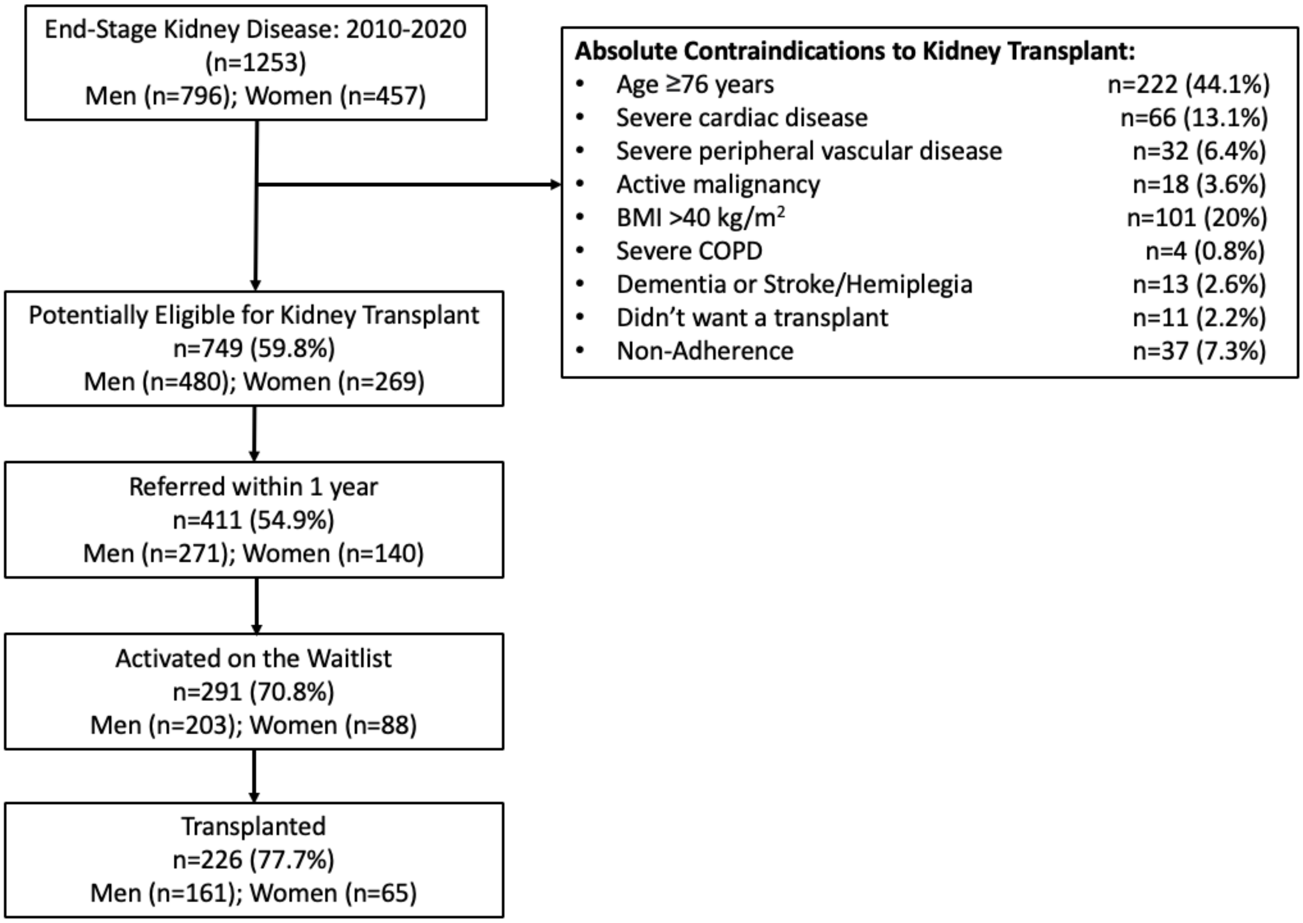

Supplemental Figure 4: Multivariable models examining gender difference (women versus men) in referral, activation, and transplantation amongst potentially eligible patients, those referred, and those waitlisted, stratified by age.

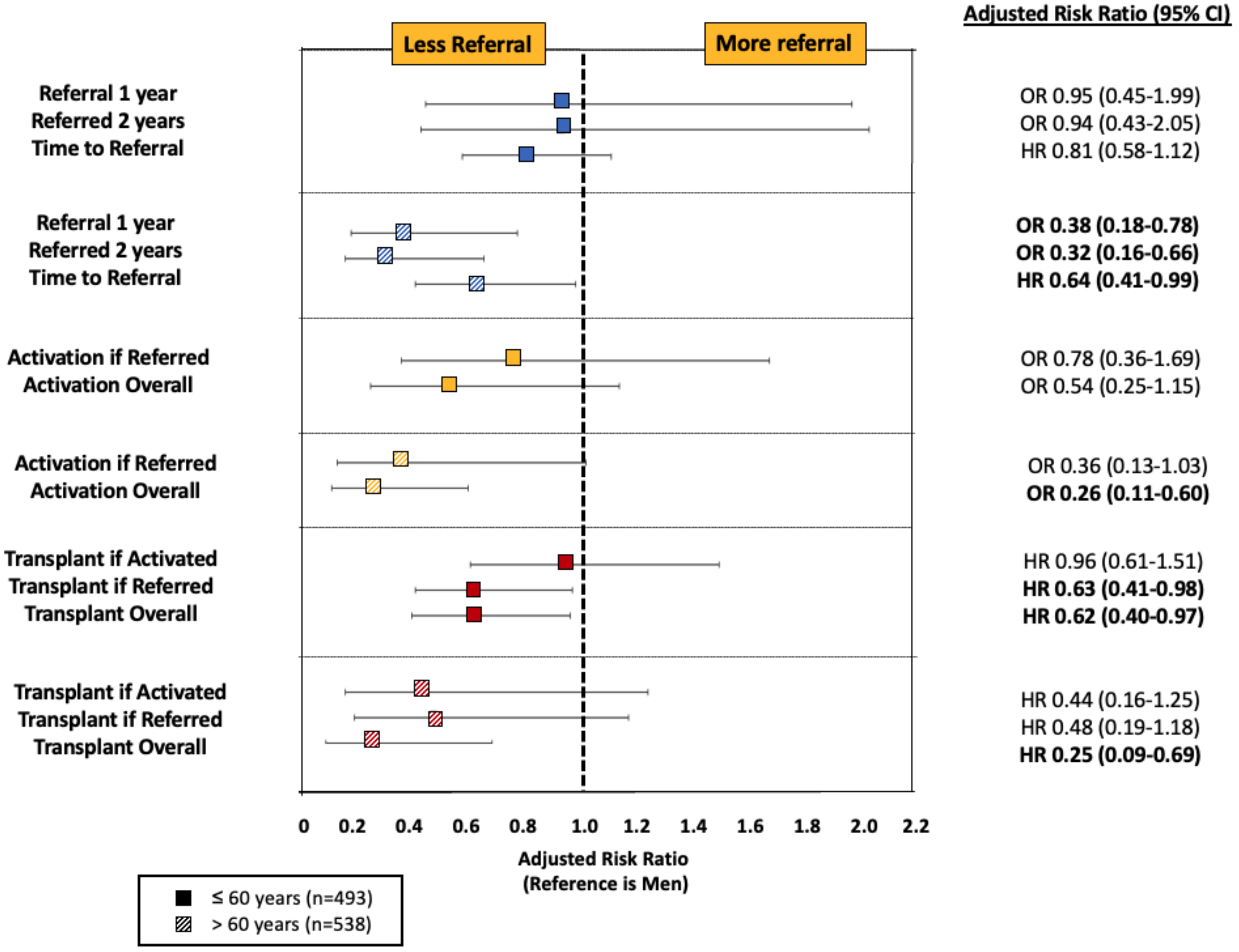

Supplemental Table 1: Multivariable logistic regression model for referral at 1 year and referral at 2 years among potentially eligible patients, activation among those referred, and multivariable Cox proportional hazards model for time to transplantation among those activated

Variable	Referral 1 year (OR)	Referral 2 years (OR)	Activation if Referred (OR)	Transplantation amongst those activated (HR)
**Gender (Women vs Men)**	0.57 (0.35-0.93)	0.53 (0.32-0.87)	0.58 (0.30-1.11)	0.74 (0.51-1.09)
**BMI >30**	1.05 (0.63-1.75)	0.96 (0.58-1.60)	0.61 (0.32-1.14)	0.83 (0.57-1.20)
**Dialysis type**				
Hemodialysis (In center)	Ref	Ref	Ref	Ref
Home Therapy (HD/PD)	3.40 (1.86-6.20)	3.85 (2.03-7.29)	1.84 (0.91-3.68)	1.22 (0.88-1.69)
**Age**				
<50	Ref	Ref	Ref	Ref
50-70	0.44 (0.22-0.89)	0.44 (0.21-0.93)	0.89 (0.40-1.99)	0.86 (0.58-1.26)
>70	0.16 (0.08-0.30)	0.17 (0.09-0.34)	0.57 (0.27-1.20)	0.59 (0.39-0.89)
**Race**				
White	Ref	Ref	Ref	Ref
Black	0.87 (0.33-2.25)	0.98 (0.38-2.51)	1.37 (0.35-5.41)	1.57 (0.75-3.31)
Other	0.96 (0.47-1.97)	0.96 (0.46-2.00)	0.32 (0.13-0.74)	1.10 (0.62-1.94)
**Cardiac Disease**	0.28 (0.16-0.50)	0.29 (0.16-0.50)	0.37 (0.16-0.87)	2.20 (1.15-4.21)
**Peripheral Vascular Disease**	0.75 (0.33-1.67)	0.56 (0.25-1.23)	1.05 (0.28-3.90)	0.30 (0.11-0.84)
**Diabetes**	0.85 (0.45-1.59)	0.88 (0.47-1.65)	0.81 (0.35-1.88)	0.73 (0.44-1.21)
**Cancer History**	0.05 (0.01-0.23)	0.04 (0.01-0.18)	0.18 (0.01-6.40)	1.56 (0.20-12.10)
**Smoker**	0.60 (0.30-1.17)	0.68 (0.36-1.29)	0.36 (0.16-0.83)	0.81 (0.46-1.43)
**Cause of ESKD**				
PCKD	Ref	Ref	Ref	Ref
Diabetes	0.18 (0.06-0.50)	0.23 (0.08-0.65)	0.50 (0.16-1.58)	0.94 (0.49-1.78)
GN	0.32 (0.12-0.87)	0.43 (0.15-1.22)	1.10 (0.40-3.04)	1.41 (0.86-2.32)
Other	0.31 (0.12-0.79)	0.31 (0.12-0.81)	0.93 (0.35-2.46)	1.27 (0.81-1.99)
**Pre ESKD Follow-up**				
Nephrologist	Ref	Ref	Ref	Ref
Specialist	1.12 (0.65-1.94)	1.31 (0.75-2.28)	0.58 (0.29-1.18)	1.00 (0.68-1.47)
Office/Clinic	2.87 (1.11-7.37)	2.29 (0.89-5.92)	0.31 (0.11-0.92)	2.07 (1.05-4.10)
No Follow-up	0.31 (0.14-0.70)	0.48 (0.22-1.06)	0.54 (0.17-1.72)	1.20 (0.66-2.16)

Supplemental Table S2a: Odds of referral at one year post-dialysis initiation in women versus men.

Variable	Men	Women
**BMI >30**	0.97 (0.51-1.85)	1.16 (0.46-3.01)
**Dialysis type**		
Hemodialysis (In center)	Ref	Ref
Home Therapy (HD/PD)	**3.88 (1.83-8.22)**	2.55 (0.82-7.88)
**Age**		
<50	Ref	Ref
50-70	**0.34 (0.14-0.84)**	0.70 (0.20-2.41)
>70	**0.19 (0.08-0.41)**	**0.10 (0.03-0.32)**
**Race**		
White	Ref	Ref
Black	0.89 (0.23-3.48)	0.57 (0.12-2.63)
Other	1.28 (0.52-3.13)	0.36 (0.10-1.31)
**Cardiac Disease**	**0.29 (0.15-0.56)**	**0.17 (0.04-0.67)**
**Peripheral Vascular Disease**	0.58 (0.23-1.44)	2.05 (0.23-18.1)
**Diabetes**	0.88 (0.40-1.91)	0.88 (0.26-2.97)
**Cancer History**	**0.04 (0.00-0.36)**	**0.02 (0.00-0.25)**
**Smoker**	0.53 (0.23-1.19)	0.50 (0.14-1.83)
**Cause of ESKD**		
PCKD	Ref	Ref
Diabetes	**0.16 (0.04-0.72)**	**0.14 (0.03-0.71)**
GN	0.26 (0.06-1.14)	**0.15 (0.03-0.73)**
Other	**0.16 (0.04-0.64)**	0.51 (0.13-2.00)
**Pre ESKD Follow-up**		
Nephrologist	Ref	Ref
No nephrologist follow-up	0.94 (0.51-1.76)	1.13 (0.42-3.01)

Supplemental Table S2b: Odds of waitlist activation amongst referred (at any time point) in women versus men.

Variable	Men	Women
**BMI >30**	0.51 (0.21-1.20)	0.76 (0.25-2.29)
**Dialysis type**		
Hemodialysis (In center)	Ref	Ref
Home Therapy (HD/PD)	2.60 (0.97-6.97)	1.48 (0.48-4.59)
**Age**		
<50	Ref	Ref
50-70	1.41 (0.44-4.53)	0.57 (0.15-2.14)
>70	0.81 (0.31-2.15)	0.30 (0.07-1.25)
**Race**		
White	Ref	Ref
Black	0.92 (0.11-7.42)	1.63 (0.16-16.4)
Other	**0.13 (0.04-0.40)**	1.62 (0.26-10.1)
**Cardiac Disease**	**0.33 (0.12-0.88)**	0.67 (0.05-9.62)
**Peripheral Vascular Disease**	0.57 (0.13-2.39)	8.12 (0.06-1134.8)
**Diabetes**	0.64 (0.20-2.09)	0.74 (0.16-3.47)
**Cancer History**	0.08 (0.00-4.20)	-
**Smoker**	0.47 (0.16-1.38)	0.24 (0.05-1.23)
**Cause of ESKD**		
PCKD	Ref	Ref
Diabetes	0.37 (0.06-2.23)	0.25 (0.03-1.93)
GN	0.75 (0.14-3.92)	0.64 (0.14-3.02)
Other	0.44 (0.09-2.24)	1.33 (0.31-5.82)
**Pre ESKD Follow-up**		
Nephrologist	Ref	Ref
No nephrologist follow-up	0.70 (0.29-1.68)	0.35 (0.09-1.32)

Supplemental Table S2c: Time to transplant amongst waitlist activated in women versus men.

Variable	Men	Women
**BMI >30**	0.77 (0.49-1.22)	1.04 (0.50-2.15)
**Dialysis type**		
Hemodialysis (In center)	Ref	Ref
Home Therapy (HD/PD)	1.06 (0.71-1.58)	0.95 (0.43-2.10)
**Age**		
<50	Ref	Ref
50-70	0.72 (0.43-1.18)	1.37 (0.63-3.00)
>70	0.67 (0.41-1.09)	0.59 (0.21-1.62)
**Race**		
White	Ref	Ref
Black	1.61 (0.64-4.09)	2.03 (0.80-8.55)
Other	1.04 (0.50-2.17)	2.62 (0.45-9.23)
**Cardiac Disease**	**2.02 (1.02-3.97)**	4.65 (0.39-55.5)
**Peripheral Vascular Disease**	**0.22 (0.06-0.80)**	0.54 (0.06-4.79)
**Diabetes**	0.80 (0.43-1.48)	0.54 (0.13-2.19)
**Cancer History**	2.37 (0.28-19.8)	-
**Smoker**	0.72 (0.37-1.39)	1.49 (0.40-5.59)
**Cause of ESKD**		
PCKD	Ref	Ref
Diabetes	1.32 (0.56-3.12)	0.51 (0.11-2.40)
GN	**2.38 (1.20-4.72)**	0.76 (0.32-1.83)
Other	1.79 (0.94-3.42)	0.91 (0.40-2.08)
**Pre ESKD Follow-up**		
Nephrologist	Ref	Ref
No nephrologist follow-up	1.16 (0.73-1.81)	0.98 (0.43-2.22)

Supplemental Table 3: Univariable logistic regression model for referral at 1 year, referral at 2 years, activation, and univariable cox proportional hazards model for time to transplantation amongst potentially eligible patients.

Variable	Referral 1 year (OR)	Referral 2 years (OR)	Activation if Referred (OR)	Transplantation amongst those activated (HR)	Transplantation amongst those referred (HR)	Transplantation amongst all potentially eligible patients (HR)
**Gender (Women vs Men)**	0.83 (0.62-1.12)	0.77 (0.57-1.04)	0.76 (0.52-1.10)	0.77 (0.56-1.05)	0.75 (0.55-1.03)	0.64 (0.46-0.87)

Supplemental Table 4: Multivariable Competing Risk Regression model (Fine and Gray) for time to transplantation amongst all potentially eligible patients, amongst those referred, and amongst those active on the waitlist.

Variable	Transplantation amongst all potentially eligible patients (SHR)	Transplantation amongst those referred (SHR)	Transplantation amongst those activated (SHR)
**Gender (Women vs Men)**	0.54 (0.36-0.81)	0.63 (0.42-0.92)	0.75 (0.50-1.11)
**BMI >30**	0.85 (0.61-1.19)	0.79 (0.57-1.10)	0.82 (0.57-1.18)
**Dialysis type**			
Hemodialysis (In center)	Ref	Ref	Ref
Home Therapy (HD/PD)	1.60 (1.15-2.23)	1.48 (1.08-2.02)	1.22 (0.88-1.68)
**Age**			
<50	Ref	Ref	Ref
50-70	0.86 (0.58-1.27)	0.92 (0.63-1.35)	0.85 (0.58-1.24)
>70	0.65 (0.43-0.97)	0.66 (0.45-0.98)	0.59 (0.39-0.89)
**Race**			
White	Ref	Ref	Ref
Black	1.28 (0.71-2.28)	1.47 (0.80-2.72)	1.59 (0.78-3.25)
Other	0.71 (0.39-1.29)	0.66 (0.37-1.18)	1.09 (0.60-1.97)
**Cardiac Disease**	1.11 (0.63-1.95)	1.18 (0.66-2.08)	2.21 (1.21-4.02)
**Peripheral Vascular Disease**	0.49 (0.19-1.26)	0.45 (0.17-1.17)	0.30 (0.10-0.89)
**Diabetes**	0.67 (0.41-1.11)	0.69 (0.43-1.11)	0.74 (0.44-1.24)
**Cancer History**	0.58 (0.16-2.07)	0.74 (0.20-2.67)	1.58 (0.90-2.77)
**Smoker**	0.73 (0.45-1.18)	0.71 (0.44-1.14)	0.81 (0.50-1.31)
**Cause of ESKD**			
PCKD	Ref	Ref	Ref
Diabetes	0.88 (0.48-1.62)	0.85 (0.47-1.54)	0.94 (0.49-1.77)
GN	1.38 (0.85-2.24)	1.45 (0.91-2.31)	1.43 (0.88-2.31)
Other	1.07 (0.70-1.64)	1.13 (0.74-1.71)	1.27 (0.85-1.90)
**Pre ESKD Follow-up**			
Nephrologist	Ref	Ref	Ref
Specialist	0.77 (0.52-1.17)	0.79 (0.54-1.17)	1.01 (0.68-1.51)
Office/Clinic	1.34 (0.65-2.75)	1.16 (0.56-2.43)	2.08 (0.98-4.44)
No Follow-up	1.15 (0.71-1.86)	1.11 (0.66-1.85)	1.20 (0.72-2.00)
**Panel Reactive Antibody**			
≤25%	Ref	Ref	Ref
>25%	1.54 (1.02-2.34)	1.27 (0.84-1.92)	0.95 (0.59-1.51)
